# Therapeutic Insights Into a Case-Control Approach to B-cell Lymphoma 3 (BCL3)-Encoded Protein by Exploring Immune Modulation and Clinical Strategies in Oral Carcinomas

**DOI:** 10.7759/cureus.83621

**Published:** 2025-05-06

**Authors:** Mostafa Ahmed Abdellah Ahmed, Lareb Asad, Madeeha Minhas, Abdul Rehman Khalil Shaikh, Seemi Tanvir, Muhmmad Hussain Shah, Aneesa Khalid

**Affiliations:** 1 General Surgery, Frimley Health NHS Foundation Trust, Frimley, GBR; 2 Pathology, Peoples University of Medical and Health Sciences, Nawabshah, PAK; 3 Health Sciences, King Saud Bin Abdulaziz University for Health Sciences, Jeddah, SAU; 4 Pathology, Liaquat University of Medical and Health Sciences, Jamshoro, PAK; 5 Pathology, Margalla Institute of Health Sciences, Rawalpindi, PAK; 6 Pathology, Dow University of Health Sciences, Dow International Medical College, Karachi, PAK; 7 Pathology, University of Health Sciences Lahore, Lahore, PAK; 8 Biomedical Sciences, Commission on Science and Technology for Sustainable Development in the South (COMSATS) University Islamabad, Islamabad, PAK; 9 Molecular Pathology and Genetics, University of the Punjab, Lahore, PAK

**Keywords:** bcl3, mucoepidermoid carcinoma, oral cancer, oral carcinoma, squamous cell carcinoma

## Abstract

Background: Oral cancer ranks as one of the most prevalent malignancies worldwide. While the role of B-cell lymphoma 3 (*BCL3*)-encoded protein as an oncogene has been explored in other epithelial cancer types, its specific contribution to oral carcinoma (OC) remains insufficiently investigated. This study investigated the expression of *BCL3* patterns in two OC subtypes and examined its influence on immune-related mechanisms.

Methodology: A cross-sectional evaluation based on a case-control study was conducted from September 2022 to January 2023, enrolling 100 participants, of whom 77 were patients diagnosed with OC and 23 were healthy control participants. Two subtypes, squamous cell carcinoma (SCC) and mucoepidermoid carcinoma (MEC), were examined. RNA was drawn from both tissue and peripheral blood samples using the QIAamp Blood RNA Kit (#51104, Qiagen, Hilden, Germany). Subsequently, *BCL3* expression was quantified using real-time PCR (RT-qPCR) with gene-specific primers. Statistical analysis was performed using one-way ANOVA via IBM SPSS Statistics for Windows, Version 20 (Released 2011; IBM Corp., Armonk, New York), with a threshold of p < 0.05.

Results and conclusion: The highest *BCL3* expression was seen in SCC samples (2.91 ± 0.62), followed by MEC samples (1.87 ± 0.58), while healthy controls showed baseline levels of expression (0.94 ± 0.49). The elevated expression of *BCL3* is highly correlated with the advanced stage of the tumour, reduced immune infiltration, and multi-modal treatment requirements. Consequently, *BCL3* appeared to put a modulatory force on the immunity environment of tumours in OC, particularly in SCC cases.

## Introduction

Oral carcinoma (OC) begins at various parts of the mouth, including the tongue, buccal mucosa, gingiva, and floor of the mouth. Squamous cell carcinoma (SCC) is considered the most prevalent type of OC [1]. OCs mainly include SCC, but there are also other types, such as mucoepidermoid carcinoma (MEC) and verrucous carcinoma, that have different characteristics. Dealing with OC presents a significant clinical challenge because patients frequently present with the disease late, and it tends to recur even after standard treatments fail [2,3]. The interest in oral carcinogenesis has grown to a level where scientists pay more attention to its molecular pathways. B-cell lymphoma 3 (*BCL3*) is a member of the nuclear IκB family. It controls the NF-κB signalling molecules to regulate immune responses, growth of tumours, and apoptosis resistance [4].

Although its functional mechanism and expression patterns during oral carcinogenesis are not well defined, research characterizes *BCL3* as a fugitive oncogene that affects a variety of epithelial and haematological malignancies [5]. The idea that elevated *BCL3* expression creates an environment where tumours lessen immune cell responses to cancers is supported by scientific data. *BCL3* may help tumours survive and progress towards malignancy by suppressing immune cells that are in charge of removing tumours [6]. *BCL3* establishes itself as a dual biomarker because it functions as an interface between cancer development and immune resistance mechanisms, which indicates therapeutic and prognostic value. Precise medicine approaches now pave the path for cancer treatment towards individualized strategies combining molecular testing and immune system examination to determine treatment selection. Medical research demonstrates that targeted treatment approaches alongside immunological drugs deliver good clinical outcomes for different cancer types [7]. The identification of reliable molecular markers, including *BCL3*, represents a vital need for OC applications because they would enable better patient stratification, treatment response prediction, and improved clinical outcomes, according to research by Umapathy et al. [8].

This study investigated the expression of *BCL3*-encoded protein in subtypes of OC (SCC and MEC) and explored its relationship with immune modulation and clinical treatment responses.

## Materials and methods

A cross-sectional analytical case-control study was conducted at Punjab University (PU) Lahore and its tertiary care hospitals from September 2022 to January 2023. The Institutional Review Board of PU Lahore approved these studies (approval no. 143/01/2022). A total of 100 participants were included in the study, 77 of whom had OC confirmed by histopathology and 23 were healthy controls with similar age and gender profiles. Using preliminary data from *BCL3* expression variance, the G*Power software version 3.1.9.4 (Heinrich Heine University Düsseldorf, Düsseldorf, Germany) was used to calculate the necessary sample size to detect an effect size of 0.25 with 80% power operating at a significance level of 0.05. Participants in the study had to be between the ages of 18 and 75. This study included new OC patients who had not received chemotherapy or radiation therapy, had not undergone surgery, and had neither acid metastases nor a second cancer. The study excluded patients with immunodeficiency conditions, chronic inflammatory disorders, autoimmune diseases, and incomplete medical records. Healthy people who lacked a history of OC or a history within their family were taken as controls. Before participating in the study, each research participant provided their written consent.

The study employed the standardized case report forms and structured interviews to gather the clinical and demographic data on participant age, gender, tumour subtype, histological grade, and cancer stage based on family history records and the American Joint Committee on Cancer (AJCC) 8th edition classification. The experts collected tumour tissues and peripheral blood samples while following the correct protocol. Total RNA was extracted from the samples using the QIAamp Blood RNA Kit (#51104, Qiagen, Hilden, Germany) according to the manufacturer's instructions. For an acceptable indicator of concentration and purity, the A260/A280 ratio of RNA samples examined using a NanoDrop 2000 spectrophotometer (Thermo Fisher Scientific, Waltham, Massachusetts) had to remain between 1.8 and 2.0. Due to the specificity of the *BCL3* gene, complementary DNA had to be produced by M-MLV reverse transcriptase (Thermo Fisher Scientific) before real-time PCR (RT-qPCR) using Applied Biosystems' SYBR Green Master Mix. Using 2^-ΔΔCt computations, GAPDH acted as the internal control to normalize expression values.

Each group underwent multiple runs of the molecular experiments to yield triple results with less than 5% variability. When performing their duties, the staff members who carried out the qPCR analysis and RNA extraction were not aware of the diagnosis status. Negative controls were run in addition to test samples that yielded Ct values greater than 35 to ensure the quality of the results. IBM SPSS Statistics for Windows, Version 20 (Released 2011; IBM Corp., Armonk, New York), was used to perform statistical analysis. Frequencies and percentages for categorical variables and average values with standard deviations for continuous variables were included in the data analysis. For every continuous data measurement, the normality distribution was assessed using the Shapiro-Wilk test. Tukey's post hoc test was used for group-wise evaluations after *BCL3* expression levels were compared between the SCC, MEC, and control groups using a one-way ANOVA. The chi-square method analyzed any categorical variables present in the study. Pearson's correlation coefficient was used to assess any relationships between *BCL3* expression levels and the progression of cancer stages. A p-value below 0.05 was considered statistically significant.

## Results

The study included 100 participants, of whom 77 (77%) were patients with OC and 23 (23%) were healthy controls with age and gender matching. The cohort was mostly made up of oral SCC (OSCC) patients (77.9%, n = 60), with MEC patients accounting for n = 17 (22.1%). Patients with OC were aged 46.4 ± 11.2 years, with a male-to-female ratio of approximately 2:1. Participants in the control group had an average age of 45.6 years, a normal distribution of 11.8 years, and an equal number of men and women. The characteristics of recruited participants are shown in Table 1.

**Table 1:** Demographic and Clinical Characteristics of the Study Participants SD: Standard Deviation; OC: Oral Carcinoma; SCC: Squamous Cell Carcinoma; MEC: Mucoepidermoid Carcinoma

Variable	OC Patients (n = 77)	Controls (n = 23)	P-value
Age (Mean ± SD)	46.4 ± 11.2	45.6 ± 11.8	0.669
Male	55 (71.4%)	15 (65.2%)	0.756
Female	22 (28.6%)	8 (34.8%)	0.745
SCC Cases	60 (77.9%)	N/A	N/A
MEC Cases	17 (22.1%)	N/A	N/A
Stage III (All Types)	46 (59.74%)	N/A	N/A
Stage IV (All Types)	31 (40.25%)	N/A	N/A

**Figure 1:**
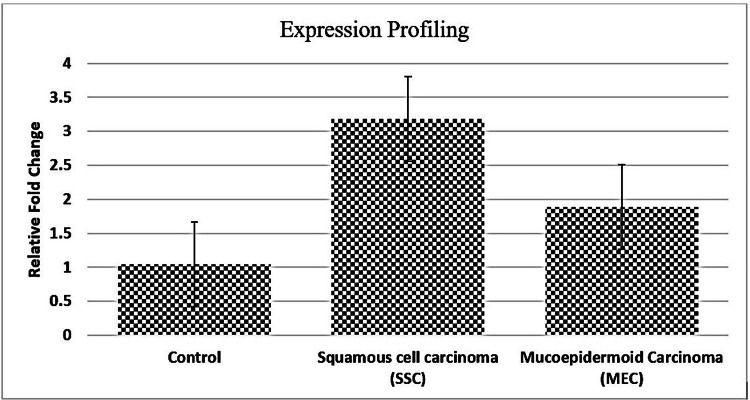
Expression Profiling

The p-values in Table 1 showed that the distribution of gender and age is not statistically significant. Quantitative RT-qPCR analysis revealed that *BCL3* expression was significantly higher in all OC case samples compared to healthy patient controls (p < 0.001). SCC tumour cells had higher expression (2.91 ± 0.62-fold) than MEC (1.87 ± 0.58-fold). Figure 1 shows that the control group had identical expression levels at baseline (0.94 ± 0.49-fold).

One-way ANOVA had demonstrated that there were significant differences in *BCL3* expression across the groups, i.e., SCC, MEC, and controls (p < 0.001). Furthermore, the classification by tumour stage revealed that *BCL3* expression was consistently elevated in Stage IV cases compared to Stage III, particularly in SCC patients. SCC showed that *BCL3* expression was 3.36 times higher than in controls, followed by another OC type, as shown in Table 2.

**Table 2:** BCL3 Expression by Subtype and Tumour Stage BCL3: B-cell Lymphoma 3

OC Subtype	Tumour Stage	Participants (%)	BCL3 Expression (Mean ± SD)	Fold Change vs. Control	P-value (Stage III vs. Stage IV)
Squamous Cell Carcinoma (SCC)	Stage III	36 (60.0%)	2.74 ± 0.56	2.91×	0.015
	Stage IV	24 (40.0%)	3.16 ± 0.66	3.36×	
Mucoepidermoid Carcinoma (MEC)	Stage III	10 (58.8%)	1.63 ± 0.45	1.73×	0.030
	Stage IV	7 (41.2%)	2.24 ± 0.61	2.38×	
Healthy Controls	N/A	23 (100%)	0.94 ± 0.49	N/A	N/A

These findings support the notion that *BCL3* overexpression correlates with the advancement of tumours and their aggressiveness. This suggests that there is potential in *BCL3* as a prognostic biomarker and a candidate for targeted immunomodulatory therapies in OC, especially SCC.

## Discussion

The study results confirmed that *BCL3* overexpression is a crucial component in the pathogenesis of OC, particularly in SCC and MEC. *BCL3* functions as an oncogene, enabling immune evasion and creating an immunosuppressive microenvironment that leads to tumour cell survival along with treatment resistance. This supports previous literature suggesting that *BCL3* facilitates immune evasion by downregulating apoptotic proteins like BAX and BAD. This downregulation suppresses pro-inflammatory cytokines as well as disrupts T-cell-mediated cytotoxicity [9]. This research showed that SCC had the highest levels of *BCL3* expression compared to MEC, but significantly reduced *BCL3* levels were observed in healthy tissue samples. Studies on cancers from various origins have proven that increased *BCL3* expression leads to aggressive tumour behaviour, reinforcing the notion of *BCL3*-mediated immunological changes as a possible driver of OC disease progression [10].

Research findings establish an important connection between *BCL3* overexpression and suppressive immune activity occurring within tumour environments. The inhibition of the pro-apoptotic proteins BAX and BAD by elevated *BCL3* expression leads to tumour cell survival and suppressed cytotoxic immune responses [11]. The immune evasion properties facilitated by this mechanism result in poor clinical outcomes and treatment resistance, particularly during advanced stages of OC. The results from this research confirm this mechanism through patient data, where higher *BCL3* expression was predominantly observed in Stage III and Stage IV tumour cases, suggesting that *BCL3* supports aggressive cancer development. Research indicates that *BCL3* generates immunosuppressive effects, aligning with studies showing that advanced disease stages correlate with poor clinical outcomes in head and neck cancers [12]. Research indicates that *BCL3* has potential in clinical prediction while also guiding individualized therapy designs.

Cases with *BCL3* overexpression may reduce the clinical success of surgical OC treatment. Patients with tumours exhibiting elevated *BCL3* expression levels require additional treatments, such as chemotherapy in combination with radiotherapy or targeted molecular drugs, to overcome treatment resistance and improve survival rates [13]. *BCL3* may serve as a primary marker to guide treatments, as it assists in customizing patient care during OCC management. Many medical studies demonstrate that *BCL3* serves as a vital oncogenic factor affecting tumour progression and immune system suppression in various malignancies, such as OSCC [14]. Some studies have confirmed that *BCL3* overexpression results in reduced treatment effectiveness and accelerated mortality among patients [15]. The current literature indicates that *BCL3* validates its ability to function as a prognostic biomarker and therapeutic target candidate for OC [16].

Further studies with functional approaches are needed to describe the precise immune response-modifying processes of *BCL3* in the tumour microenvironment, based on gene expression levels. Research should focus on verifying the effectiveness of *BCL3*-targeted therapy while also conducting extended studies to evaluate how *BCL3* inhibition impacts OC clinical outcomes. Despite its strengths, this study has several constraints that researchers should note when interpreting the results. The observational nature of the study design prevents researchers from concluding that OC progression is indeed a result of *BCL3* overexpression.

## Conclusions

The research demonstrated that *BCL3* functions as a vital biological marker affecting the development of OC. In addition to immune suppression and advanced tumour stages, the study identified directly correlating variables related to elevations in *BCL3* expression. The outlook for *BCL3* as a prognostic marker for OC has been confirmed, and its potential integration with both surgical approaches and precision medicine treatments suggests new possibilities for individualized therapy specifically for OC.

Further research on *BCL3* inhibitor development and immune system modulation therapies is necessary to establish effective treatments for patients with OC.
